# Visual, Topographic and Aberrometric Outcomes After Phototherapeutic Keratectomy (PTK) for Salzmann Nodular Degeneration

**DOI:** 10.3390/medsci13030197

**Published:** 2025-09-18

**Authors:** Simon Helm, Johanna Wiedemann, Niklas Reinking, Benjamin Rosswinkel, Björn Bachmann, Claus Cursiefen, Simona Schlereth

**Affiliations:** 1 Department of Ophthalmology, Faculty of Medicine and University Hospital Cologne, 50931 Cologne, Germany; 2 Reinking Augenklinik am Campus, 33100 Paderborn, Germany; 3 Institute of Medical Statistics and Computational Biology, Faculty of Medicine and University Hospital Cologne, University of Cologne, 50923 Cologne, Germany; 4 Department of Ophthalmology, Center for Molecular Medicine Cologne, Faculty of Medicine and University Hospital Cologne, University of Cologne, 50923 Cologne, Germany

**Keywords:** phototherapeutic keratectomy, Salzmann nodular degeneration, higher order aberration, densitometry, astigmatism

## Abstract

**Purpose:** The aim of this paper is to study the visual outcomes, changes in higher order aberration (HOA) and corneal densitometry after debridement and excimer laser phototherapeutic keratectomy (PTK) for the treatment of Salzmann nodular degeneration (SND). **Methods:** This monocentric study includes 69 eyes from 54 patients who underwent debridement and PTK for SND (mean follow-up time of 447.1 ± 597.7 days post-operatively). The following parameters were measured before and after PTK: best corrected visual acuity (BCVA) in logMAR, sphere, cylinder, calculated spherical equivalent (SPHQ), mean and maximum refractive power, astigmatism, HOA, corneal density and thickness. Patients were divided into two cohorts depending on additional visual acuity limitations (VAL). **Results:** Mean visual acuity improvement was 0.16 ± 0.21 logMAR (*p* < 0.001), independent of additional VAL, and was associated with normalization of the cornea (hyperopic reduction by 2.13 ± 2.60 dpt, *p* < 0.001), reductions in cylinder (1.49 ± 2.44 dpt, *p* < 0.001) and corneal astigmatism (3.01 ± 3.39 dpt, *p* < 0.001). HOA was reduced by 0.77 ± 1.11 µm (*p* < 0.001) and corneal density by 6.08 ± 16.45 gray scale units (GSUs) in the center (*p* = 0.019) and by 9.32 ± 12.08 GSUs in the mid-periphery (*p* < 0.001). Haze occurred in 26.1% of patients (15.9% mild; 10.1% moderate). Re-PTK was necessary in 5.8%. **Conclusions:** PTK is a low-complication method for visual improvement in patients with SND, regardless of additional VAL, and is associated with a normalization of corneal parameters. HOA, corneal density and K_max_ were reduced significantly and showed a correlation with visual acuity, implying that these objective parameters may have a good predictive value for visual acuity.

## 1. Introduction

As the first layer of the transparent tissue of the eye, the cornea is responsible for 2/3 of the refractive power of the eye. Its shape significantly determines the refractive power, so an irregular shape due to scarring and nodular deposits, such as in degenerative and hereditary corneal diseases, can cause a blurred and distorted projection on the retina along with pain and foreign body sensation [1,2,3,4]. Phototherapeutic keratectomy (PTK) has been used for many years in the treatment of corneal degenerations and dystrophies and, through the use of an excimer laser, enables high-precision removal of deposits and opacities [2,5].

Salzmann nodular degeneration (SND) was first described in 1925 as a disease of the cornea in which subepithelial, hyaline nodules and opacities of the cornea occur [2], which lead to irregular thickness of the cornea combined with irregular astigmatism [2,6]. SND can be asymptomatic or lead to a reduction in visual acuity and a foreign body sensation. The bluish, light gray opacity of the cornea can be multifocal or isolated. The opacities occur more frequently peripherally than centrally [2]. SND is more common in middle-aged women, is bilateral in over 50% of cases and is often associated with chronic ocular surface inflammation such as meibomian gland dysfunction, dry eye or keratoconjunctivitis [4]. Even though SND is considered a degenerative disease, familial clusters are observed [2].

Treatment options for SND include conservative management, mechanical debridement (superficial keratectomy) and excimer laser PTK. While debridement alone can provide symptomatic relief, recurrence rates of up to 22% have been reported [2,5]. In contrast, PTK in combination with mechanical debridement allows for more complete removal of nodular and subepithelial material, often resulting in better topographic regularity and lower recurrence, particularly when combined with mitomycin C [5,7]. However, objective data on structural and functional changes after PTK in SND—such as higher-order aberrations (HOAs) and corneal densitometry—remain limited in the literature.

The aim of this retrospective study was to investigate the outcome of mechanical debridement followed by PTK in the treatment of SND in a large patient cohort and to contribute knowledge about the effects of treatment on visual acuity, refractive parameters, HOA and corneal densitometry.

## 2. Methods

This monocentric, retrospective study was performed at the Department of Ophthalmology of the University Hospital Cologne, Germany. The study was conducted in accordance with the Declaration of Helsinki and reviewed by the Ethics Committee of the University of Cologne. Due to its retrospective nature, it was not deemed subject to approval.

### 2.1. Inclusion and Exclusion Criteria

**Figure 1:**
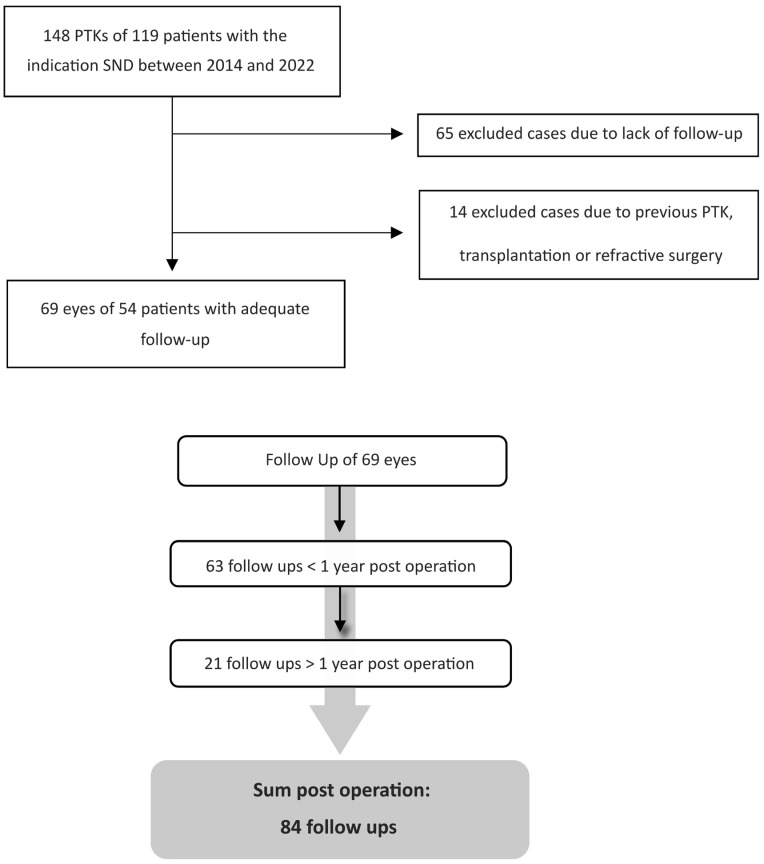
Consort flow diagram showing the collection of the clinical data. Main inclusion criteria was PTK due to SND. For statistical analysis differentiation was made whether follow-up was <1 year or >1 year post-operatively. We documented 63 follow-ups <1 year and 21 follow-ups >1 year after PTK. In total, 84 follow-up visits were recorded across 69 eyes. For statistical analysis in the “total” follow-up group (69 eyes), only the latest available follow-up for each eye was analyzed.

Only patients who underwent PTK for SND were included (Figure 1). The pre- and post-operative data (2.2 Recorded Data) of 69 eyes from 54 patients were included in this study. There was no uniformly defined follow-up period. Based on the time interval between the follow-up examinations and the surgery, follow-ups were divided into groups <1 year and >1 year. In the “total” group, only the most recent follow-up was considered for each eye (Figure 1).

The number of eyes included in each analysis varied depending on data availability for specific parameters (e.g., HOA, densitometry, refractive values).

Patients were excluded if they had already undergone PTK, corneal refractive surgeries, received a corneal transplant or did not present themselves to the Department of Ophthalmology of the University Hospital Cologne for further follow-up examinations.

### 2.2. Recorded Data

We analyzed best corrected visual acuity (BCVA) (logMAR), sphere, cylinder, spherical equivalent (SPHQ, dpt), refractive power K_m_ and K_max_ (dpt), higher order aberration (HOA, µm), densitometry (gray scale unit (GSU)), astigmatism (dpt) and pachymetry (µm) before and after PTK. For densitometry, the range was 0 GSU (maximum transparency) to 100 GSU (completely opaque cornea) [8,9,10].

Additionally, we analyzed the results of BCVA both independently and dependent on additional visual acuity limitations (VAL). VAL were defined as any disease of the eye, which lead to a decrease in visual acuity which could not explained only by the SND itself. The relevance of VAL was determined by experienced senior physicians (Table 1).

Complications and ablation depth were also evaluated. The data were extracted from the electronic data processing program Orbis (Dedalus Health Care, Bonn, Germany) and Fidus (Arztservice Wente, Darmstadt, Germany) and collected in Microsoft Excel (Redmond, WA, USA).

### 2.3. Pre-Operative Procedure

All patients underwent pre-operative clinical examination of the eyes, which included a slit lamp examination and intraocular pressure, visual acuity and refraction determination (subjective and by an autorefractometer), examination of the fundus, anterior segment optical coherence tomography (OCT), macular OCT and Scheimpflug Imaging with the Pentacam HR Type 70.900 (Oculus GmbH, Wetzlar, Germany). The indication for PTK was based on the subjective disturbance caused by foreign body sensation, higher corneal astigmatism, or a reduction in visual quality due to nodular deposits, clouding, or scarring of the cornea due to SND.

### 2.4. Surgical Procedure

Anterior-segment OCT was used to determine the depth of the lesion. The patients were pre-treated with local anesthesia and disinfection and then placed in a lying position under a Schwind Amaris 750S excimer laser. First, the epithelium and Salzmann nodes were removed using a hockey knife. The laser was then used to remove any residual opacities and with the use of masking fluids. This was followed by a localized treatment with 0.02% mitomycin for 15 s. Mitomycin was thoroughly washed off and a bandage lens was inserted. This was left in place until epithelial closure was confirmed. Follow-up treatment was carried out with local antibiotic eye drops (ofloxacin) until epithelial closure and tear substitutes. Topical steroids (dexamethasone) were administered four times daily for four weeks and tapered over a period of four weeks.

Patients with blepharitis were instructed to perform lid margin hygiene before and after the surgery. Demodex was treated pre-operatively, in cases of demodex contamination.

### 2.5. Statistical Test Procedure

All tests were performed using IBM SPSS Statistics (version 29.0.1.0; Chicago, IL, USA). Before the paired *t*-test was applied, the difference between the post- and pre-PTK data was checked for a normal distribution with a Shapiro–Wilk test. A normal distribution was assumed for groups with n ≥ 30. If a normal distribution was violated, the Wilcoxon non-parametric test was used. For parameters with the expected direction, one-sided p was used. In case of spheres, cylinders, SPHQ, K_m_ and K_max_ two-sided p was used because of an unclear direction of change. Bivariate correlation with the Pearson-coefficient was used to analyze a possible correlation.

## 3. Results

### 3.1. Descriptive

This study included 69 eyes from 54 patients (Table 1). The mean age was 61.6 ± 14.5 years and 72.0% (n = 49) of the included eyes were from female patients. Additional visual limitations were present in 44.9% (n = 31) of the patients. The most common visual acuity limitation (VAL) was cataract (36.2%). The mean follow-up time for the <1 year group was 134.0 d ± 108.9, for >1 year was 1179.7 d ± 617.1 and for the most recent follow-up (total) was 447.1 d ± 597.7.

### 3.2. BCVA (logMAR) with and Without Additional VAL

**Figure 2:**
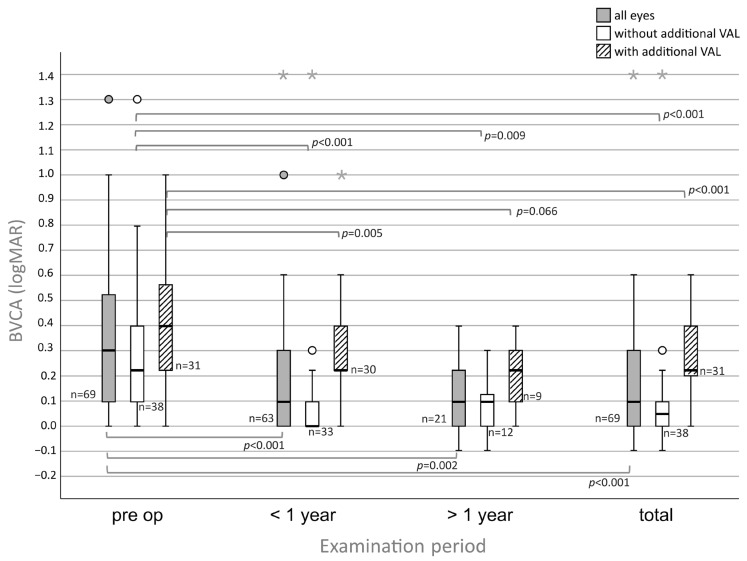
Improvements in visual acuity for eyes with SND undergoing PTK: If analyzed independently of additional visual acuity limitations (VAL) (gray boxplot), all eyes show significant improvements in all follow-ups (*p* < 0.001, *p* = 0.002, *p* < 0.001). If analyzed dependent on additional VAL, eyes without additional VAL (green boxplot) show significant improvements in visual acuity in all follow-ups as well (*p* < 0.001, *p* = 0.009, *p* < 0.001). Eyes with additional VAL (red boxplot) improved significantly except in the follow-up >1 year (*p* = 0.005, *p* = 0.066, *p* < 0.001) Circles (°) indicate mild outliers (>1.5 × IQR and ≤3 × IQR), asterisks (*) indicate extreme outliers (>3 × IQR).

**Figure 3:**
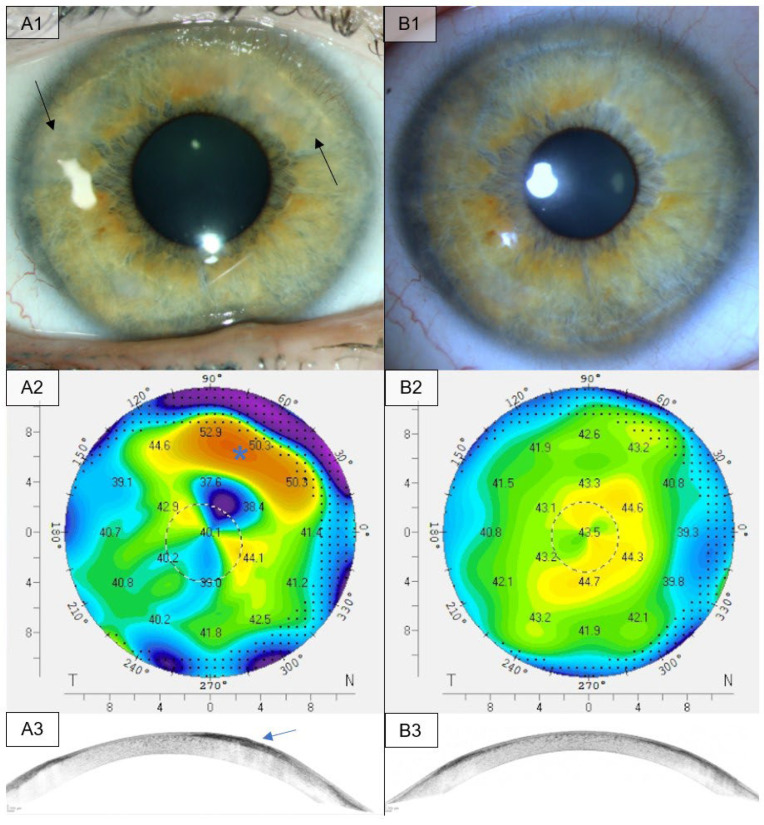
Slit lamp, Pentacam and anterior segment OCT examination of a patient suffering SND before and after PTK. (**A1**): Pre-operative slit lamp examination showing multifocal subepithelial hyaline nodules in the upper mid-periphery leading to large areas of opacity in the cornea (black arrows). (**A2**): Pre-operative Pentacam examination with a color map of the frontal corneal curvature showing deviations in the regions of the nodules (blue asterisk). (**A3**): Pre-operative anterior segment OCT showing the SND-nodule (blue arrow). (**B1**): Post-operative slit lamp examinations three years after PTK. Visual acuity improved from 0.22 logMAR pre-operatively to 0.10 logMAR post-operatively. (**B2**): The post-operative Pentacam examinations show a normalization of the frontal corneal curvature. (**B3**): Post -operative OCT examination shows a restoration of the corneal surface in the region of the resected nodule.

Analyzing all eyes, including patients with and without VAL, the pre-operative BCVA (mean ± SD; logMAR) was 0.34 ± 0.28 (n = 69) (Figure 2 and Figure 3). At follow-up <1 year post-operatively, the BCVA increased to 0.21 ± 0.25 (*p* < 0.001, n = 63). At follow-up >1 year post-operatively, the BCVA was 0.13 ± 0.14 (*p* = 0.002, n = 21). In the total group, the BCVA improved up to 0.18 ± 0.23 (*p* < 0.001, n = 69).

In the subgroup analysis of the cohort without VAL, the pre-operative BCVA was 0.26 ± 0.28 (n = 38). At follow-up <1 year, the BVCA improved up to 0.11 ± 0.25 (*p* < 0.001, n = 33). At follow-up >1 year, the BCVA improved up to 0.08 ± 0.11 (*p* = 0.009, n = 12). In the total group, BVCA improved up to 0.10 ± 0.24 (*p* < 0.001, n = 38).

Sub-analyzing the cohort with additional VAL, pre-operatively the BCVA was 0.43 ± 0.26 (n = 31) (Figure 2). At follow-up <1 year, BCVA improved up to 0.32 ± 0.21 (*p* = 0.005, n = 30). At follow-up >1 year, the BCVA improved up to 0.20 ± 0.15 (*p* = 0.066, n = 9). In the total group, BCVA improved to 0.27 ± 0.17 (*p* < 0.001, n = 31).

### 3.3. Refraction

**Figure 4:**
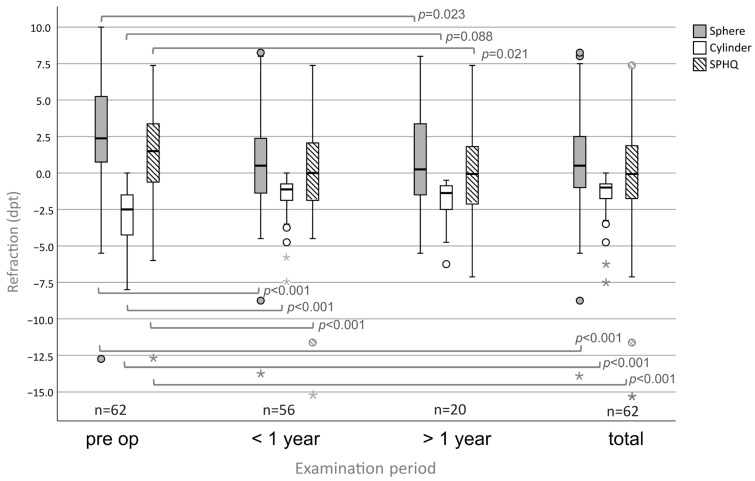
Refraction in eyes with SND undergoing PTK: significant changes in all follow-ups except for the negative cylinder > 1 year.) Circles (°) indicate mild outliers (>1.5 × IQR and ≤3 × IQR), asterisks (*) indicate extreme outliers (>3 × IQR).

Pre-operatively, the sphere (mean ± SD; dpt) was +2.78 ± 3.78 (n = 62) (Figure 4). At follow-up <1 year, the sphere was +0.72 ± 3.85 (*p* < 0.001, n = 56). At follow-up >1 year, the sphere was +0.83 ± 3.80 (*p* = 0.023, n = 20). In the total group, the sphere was 0.65 ± 3.87 dpt (*p* < 0.001, n = 62).

The cylinder (mean ± SD; dpt) was −3.11 ± 2.11 dpt (n = 62) pre-operatively. At follow-up <1 year, the cylinder was −1.54 ± 1.43 (*p* < 0.001, n = 56). At follow-up >1 year, the cylinder was −1.84 ± 1.56 (*p* = 0.088, n = 20). In the total group, the cylinder was −1.62 ± 1.52 (*p* < 0.001, n = 62).

The spherical equivalent (SPHQ) (mean ± SD; dpt) was 1.22 ± 3.43 (n = 62) pre-operatively. The SPHQ was −0.05 ± 3.87 at follow-up <1 year after PTK (*p* < 0.001, n = 56). At the follow-up > 1 year after PTK the SPHQ was −0.09 ± 3.72 (*p* = 0.021, n = 20). In the total group the SPHQ was −0.16 ± 3.87 (<0.001, n = 62).

### 3.4. K_m_ and K_max_

**Figure 5:**
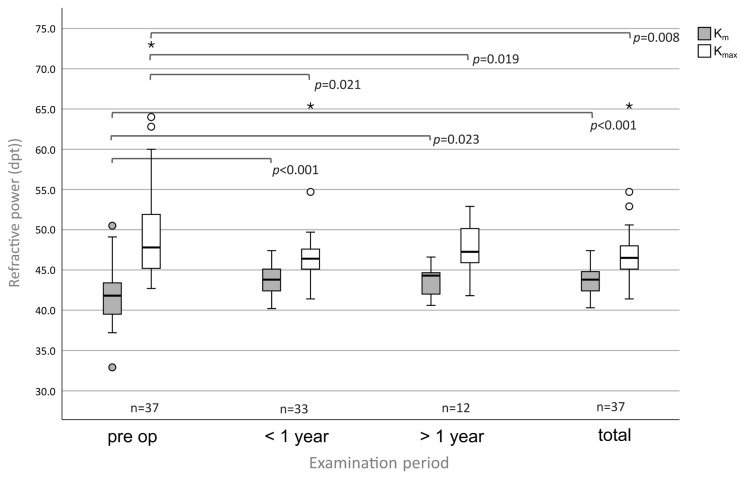
Keratometric power indices K_m_ and K_max_ show significant changes in all follow-ups in eyes with SND undergoing PTK.) Circles (°) indicate mild outliers (>1.5 × IQR and ≤3 × IQR), asterisks (*) indicate extreme outliers (>3 × IQR).

The pre-operative K_m_ and K_max_ (mean ± SD; dpt), measured by Pentacam, were 41.80 ± 3.34 (n = 37) and K_max_ 50.04 ± 6.83 (n = 37), respectively (Figure 5). At follow-up <1 year the K_m_ increased up to 43.77 ± 1.85 (*p* < 0.001, n = 33) and the K_max_ decreased to 46.95 ± 4.19 (*p* = 0.021, n = 33). At follow-up >1 year the K_m_ increased up to 43.75 ± 1.82 (*p* = 0.023, n = 12) while the K_max_ decreased to 43.75 ± 1.82 (*p* = 0.019, n = 12). In the total group the K_m_ was 43.75 ± 1.81 (*p* < 0.001, n = 37) and the K_max_ 47.22 ± 4.15 (*p* = 0.008, n = 37).

### 3.5. Astigmatism, HOA and Pachymetry

Pre-operatively, the corneal astigmatism (mean ± SD; dpt), measured by Pentacam, was 4.49 ± 3.56 dpt (n = 37) (Table 2). At follow-up <1 year post-operatively, the corneal astigmatism decreased to 1.32 ± 1.19 (*p* < 0.001, n = 33). At follow-up >1 year, the corneal astigmatism decreased to 1.92 ± 1.24 (*p* = 0.034, n = 12). Regardless of follow-up duration, the entire post-operative corneal astigmatism decreased to 1.49 ± 1.20 (*p* < 0.001, n = 37).

The pre-operative HOA (mean ± SD; µm) was 1.32 ± 1.17 (n = 35). At follow-up <1 year, the HOA decreased to 0.52 ± 0.49 (*p* < 0.001, n = 31). At follow-up >1 year, the mean HOA was 0.53 ± 0.40 (*p* = 0.005, n = 10). The total post-operative HOA was 0.55 ± 0.50 (*p* < 0.001, n = 35).

The corneal thickness at the center and thinnest point of the cornea (mean ± SD; µm) were compared. Pre-operatively, the thickness at the center was 568.95 ± 46.01 (n = 37) in the center and 550.00 ± 47.86 (n = 37) at the thinnest point. The mean ablation depth of the laser was 18.0 µm (±6.9, n = 55).

At follow-up <1 year, the thickness at the center was 533.55 ± 31.53 (*p* < 0.001, n = 33) and 527.15 ± 31.61 at the thinnest point (*p* < 0.001, n = 33). At follow-up > year, the thickness at the center was 533.17 ± 31.94 (*p* = 0.007, n = 12) and 526.83 ± 33.80 at the thinnest point (*p* = 0.058, n = 12). In the total group, the corneal thickness decreased in the center to 535.11 ± 30.47 (*p* < 0.001, n = 37) and at the thinnest point to 528.03 ± 31.66 (*p* < 0.001, n = 37).

### 3.6. Densitometry

**Figure 6:**
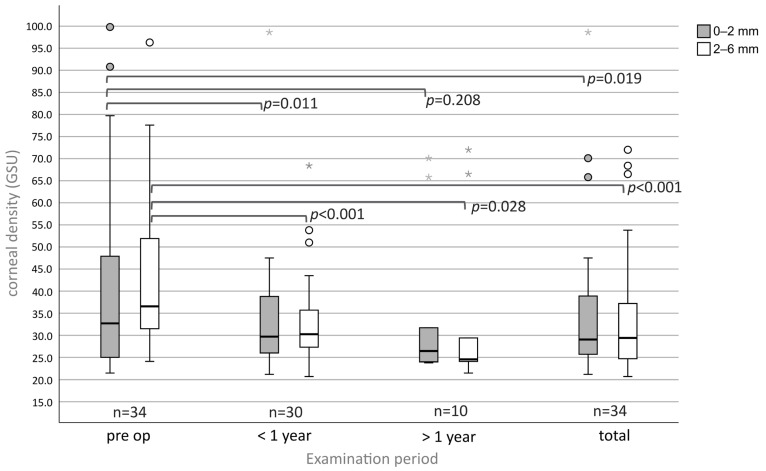
Eyes with SND undergoing PTK show a significant reduction in the corneal density (GSU) in the center and in the periphery except in the center at the follow-up > 1 year. Circles (°) indicate mild outliers (>1.5 × IQR and ≤3 × IQR), asterisks (*) indicate extreme outliers (>3 × IQR).

Densitometry was measured in the 0–2 mm (center) and 2–6 mm (mid-periphery) zones (mean ± SD) and was described in standardized grayscale units (GSU) (Figure 6).

Pre-operatively, the corneal density was 41.11 ± 21.28 (n = 34) in the center and 43.63 ± 17.50 (n = 34) in the mid-periphery. At follow-up <1 year, the values decreased significantly to 33.57 ± 14.37 (*p* = 0.011, n = 30) and 32.99 ± 10.21 (*p* < 0.001, n = 30). At follow-up >1 year (n = 11), the central density was 34.64 ± 17.75 (*p* = 0.208, n = 10) and 33.85 ± 18.85 (*p* = 0.028, n = 10) in the mid-periphery. In total, the density decreased in the center to 35.03 ± 15.99 (*p* = 0.019, n = 34) and in the mid-periphery to 34.31 ± 13.30 (*p* < 0.001, n = 34).

### 3.7. Correlations

Before and after PTK there was a significant, linear correlation between the parameters HOA, densitometry and K_max_ with visual acuity (logMAR) (Table 3).

### 3.8. Complications

The complication rate (Table 4) was determined based on visit entries at follow-up. Terms such as “minimal”, “light”, “fine” or similar were interpreted as mild findings. Terms such as “pronounced”, “distinct”, “large” or similar, on the other hand, were considered advanced findings. Findings without other attributes were classified as moderate. Haze was mainly found in the area underneath the Salzmann nodules, and was therefore mainly located at the periphery. Mild haze was seen in up to 15.9%, whereas strong haze was not seen at all. Dry Eye was seen in up to 18% of patients, and this was often mild.

In two eyes of two patients (2.9%), a re-PTK was necessary in the post-operative course due to residual deposits and insufficient visual improvement (at 1.5 and 11.0 months after the initial PTK). Another two eyes of two patients (2.9%) required a re-PTK due to recurrence at 2.5 and 5.5 years after the initial PTK.

## 4. Discussion

Our study showed that PTK is an effective method for improving visual acuity in patients with SND regardless of additional VALs and adds new information about changes in HOA and corneal densitometry in a large cohort (n > 30) with a relatively long follow-up period. This study is the first to measure the effects of PTK on these parameters.

In this study, we observed a significant reduction in HOA and densitometry at all follow-up periods. Furthermore, a correlation of HOA, densitometry, and K_max_ with the visual acuity before and after PTK was observed. This suggests that they are reliable improvement parameters and helps verify the therapeutic success of PTK on an objective level, since a subjective improvement in visual acuity can be delayed and may also be influenced by additional VAL, e.g., cataract, glaucoma or retinopathy. We also demonstrated a significant myopic shift in patients with SND after PTK and confirmed the results and tendencies shown in previous studies with a larger cohort and longer follow-up times [5,7,11].

The improvement of BCVA in the cohort without VAL to 0.16 ± 0.20 logMAR and in the cohort with additional VAL to 0.16 ± 0.23 logMAR, suggest that despite worse initial visual acuity (0.43 ± 0.26 logMAR vs. 0.26 ± 0.28 logMAR), patients with additional VAL could benefit from PTK in almost the same way as patients without VAL. If analyzed independently of existing VAL, an improvement of 0.16 ± 0.21 logMAR (*p* < 0.001) over the entire course is shown. This is almost identical to the result in a study by Mahler et al., where the Amaris 750S (Schwind) laser achieved an improvement in visual acuity of 0.17 ± 0.33 logMAR (*p* < 0.013) [11]. A similar improvement after PTK in SND has been previously described by Das, Seitz, and Khaireddin et al. [5,7,12,13]. Our data suggest that PTK should be offered to patients with SND irrespective of other visual limitations.

Hyperopic, irregular astigmatism is typical in SND [2]. Nodes in the mid-periphery led to “asymmetric tear film pooling” with consecutive “optical cornea plana” [11,14]. The removal of the nodules eliminates this hyperopic effect and leads to an improvement in corneal regularity, as can be seen in our study [2,11]. Here, a significant shift from hyperopia towards emmetropia by 1.38 dpt (±1.83) SPHQ (*p* < 0.001, n = 62) can be observed. Mahler et al. did not observe a significant shift in subjectively measured refraction. Only the objectively measured SPHQ changed significantly from 1.92 ± 2.37 dpt pre-operatively to −1.43 ±3.15 dpt post-operatively after 6 months (*p* < 0.001, n = 18) [11]. Das et al. also demonstrated a myopic shift of 1.5 ±0,7 dpt (n = 14), as did Khaireddin et al. (n = 8) [5,7]. We now confirm these earlier study results in a larger cohort involving 69 eyes (full refractive data were available for 62 patients). The subjectively measured refractive change was also reflected in the analysis of the refractive indices by Pentacam. We observed that K_m_ increased by 1.95 dpt (±2.74) (*p* < 0.001, n = 37) which represents the normalization of the corneal surface. At the same time K_max_ decreased by 2.82 dpt (±6.07) (*p* = 0.008, n = 37) which can be explained by the fact that areas with extreme refractive values in the area of Salzmann’s nodules were removed after PTK.

As expected, patients with SND benefited from a highly significant improvement in topographic astigmatism of 3.01 dpt (±3.39) (*p* < 0.001, n = 37). The subjectively measured cylinder also improved significantly by 1.49 dpt (±2.44) in the present study (*p* < 0.001, n = 62). Our study, with a larger cohort and longer follow-up time, confirms and builds upon the results of earlier studies. Das et al. demonstrated that the negative cylinder improved from 1.0 to 0.7 dpt (n = 14) [5]. Mahler et al. showed a significant improvement in topographic astigmatism by 1.73 ± 1.99 dpt (Schwind) and by 1.99 ± 2.21 dpt (Zeiss). On the other hand, in their study, no significant reduction in the subjective cylinder was observed [11].

HOA was reduced by a mean of 0.77 µm (±1.11) (*p* < 0.001, n = 35). These data confirm the results of study by Reddy et al. (n = 13) with a larger cohort [15].

Densitometry can be used as an objective parameter for the (quantitative) assessment of corneal opacities, which, along with distorted and blurred vision, can be responsible for poor visual acuity [8,16]. As expected, PTK can help normalize corneal density significantly. We observed a stronger decrease peripherally (9.32 ± 12.08 GSU) than centrally (6.08 ± 16.45 GSU), fitting the clinical findings [11,17]. At the time of publication, there are no other studies that have measured corneal density before and after PTK in SND.

A normal corneal density should be around 22 GSU in the center and 21 GSU in the mid-periphery, as shown by a study with 395 eyes [16]. PTK led to a normalization in corneal densitometry, although the post-operative values for SND in the center of 35.03 ± 15.99 GSU and in the periphery of 34.31 ± 13.30 GSU still deviate from healthy controls.

In summary, corneal density can be reduced significantly after PTK, indicating that visual acuity in SND was reduced by an increase in corneal density, and not only due to corneal astigmatism.

Significant changes in the thickness at the center and thinnest part of the cornea (*p* < 0.001) were observed. The values correspond to those of a healthy cornea of 533 ± 53 μm. [18].

The average ablation of the laser was 18.0 μm (±6.9, n = 55) which is comparable with the results of Mahler et al. [11]. Lee at al. used a deeper ablation of 41 ± 43 μm [19]. The mean reduction in thickness in this study is 33.84 µm (±33.85) at the center and 21.97 µm (±31.99) at the thinnest point (*p* < 0.001, n = 37) due to the removal of Salzmann nodules and tissue.

Significantly reduced HOA, densitometry (centrally and mid-peripheral), and K_max_ all showed a significant positive correlation with visual acuity (logMAR) before and after PTK. Without establishing a causal link, these results can be reconciled with the hypothesis that the improvement in visual acuity can be attributed to the removal of corneal opacity (densitometry), restoration of the original uniform corneal shape (HOA) and removal of areas with extreme refractive power (K_max_). Thus, these values can reliably record the success of therapy objectively.

In SND mild haze occurred in 15.9% of eyes (n = 11) and moderate haze in 10.1% (n = 7) after PTK, predominantly in areas previously affected by Salzmann nodules, which were mainly located peripherally. In four eyes of two patients (11.8%), intensified corticosteroid therapy was necessary to resolve the haze. This pattern is consistent with our densitometry results, which showed a significantly greater reduction in the mid-periphery (−9.32 ± 12.08 GSU) compared to the central zone (−6.08 ± 16.45 GSU).

Three eyes (4.4%) required a re-PTK due to SND residuum and lack of improvement in visual acuity and one eye needed a re-PTK due to a SND-recurrence (1.6%). Overall, the recurrence rate in our cohort (5.8%) was lower than in a previous long-term series using a similar surgical approach combining mechanical nodule removal with PTK. In that study, morphologic recurrence occurred in approximately 31% of eyes, and 18% required repeat PTK due to visually significant recurrence [5]. These discrepancies may be related to differences in accompanying procedures, such as the intraoperative use of mitomycin C, strict pre-operative treatment of blepharitis, as well as differences in cohort size and follow-up duration. Larger series such as the present study are likely to provide more stable recurrence estimates.

In addition to the combined approach of mechanical debridement and PTK, SND can also be managed by superficial keratectomy alone. While this may be sufficient in selected cases, recurrence rates of up to 22% have been reported following debridement without laser ablation [2]. In contrast, combined treatment with PTK—especially when performed with adjunctive mitomycin C—has been associated with lower recurrence rates and more consistent improvements in visual acuity [5,7]. Furthermore, PTK contributes to the normalization of the corneal surface and reduction in higher-order aberrations, which may improve functional outcomes beyond what is achievable with debridement alone [15]. These findings support the combined approach as a more effective and durable treatment option for SND in cases with central involvement.

Various factors may have led to bias in this study. A major problem was the follow-up of patients: many patients could not be included in the study because they did not receive follow-up care at the University Hospital of Cologne (UHC). Patients may be sent from distant areas and therefore have to travel long distances for treatment. In such cases, follow-up examination was carried out by a local ophthalmologist near the place of residence. Only 69 of 148 eyes (47%) were examined after PTK at the UHC and were included in the study. It is also possible that patients chose to present at the UHC for follow-up as a result of subjective complaints, which may contribute to selection bias. However, complications are rare, and all patients were asked to return to our clinic if they had any complaints. Additionally, the time of follow-up was not standardized which may also contribute to study bias.

## 5. Conclusions

With the help of PTK, improvement in visual acuity can be achieved with minimal complications in almost all patients with SND. The formation of peripheral nodules in SND can lead to flattening of the central cornea, which can in turn cause hyperopia [5,11,14,20]. By eliminating the nodules and the resulting “optical cornea plana” [13,14], the normalization of the corneal surface can be achieved. The consecutive myopic shift and the reduction in subjective and objective astigmatism was demonstrated in our study with a larger cohort and long follow-up. Additionally, restoration of the original corneal shape and removal of corneal opacities was observed by the objective parameters of HOA and densitometry. In this regard, our study contributes further data which showed a significant reduction in HOA and densitometry after PTK. Furthermore, the correlation of HOA, densitometry, and K_max_ with visual acuity before and after PTK proved PTK to be an effective therapy on an objective level, promising a good predictive value for visual outcomes.

## Figures and Tables

**Table 1:** Mean follow-up time in days (d) and descriptive results of the patients with SND undergoing PTK showing age, sex, additional visual acuity limitations, previous eye surgeries and ablation depth of the excimer laser.

**Mean follow-up time in days (d)**	
<1 year	134.0 d (±108.9, n = 63) min: 4 d, max: 364 d
>1 year	1179.7 d (±617.1, n = 21) min: 381 d, max: 2280 d
Total	447.1 d (±597.7, n = 69) min: 5 d, max: 2280 d
Female	71.0% (n = 49)
Male	29.0% (n = 20)
Age at intervention (y)	61.6 (±14.5, n = 69)
**Visual acuity limitations (VAL):**	
None	55.1% (n = 38)
One or more: Distribution of Visual limitation:	44.9% (n = 31)
Cataract	36.2% (n = 25)
Keratitis sup. punctate	7.3% (n = 5)
Glaucoma	7.2% (n = 5)
Retinopathy	5.8% (n = 4)
Optic neuropathy	1.4% (n = 1)
Fuchs Endothelial Dystrophy	4.3% (n = 3)
Amblyopia	2.9% (n = 2)
**Previous eye surgery due to other eye diseases**:	20.3% (n = 14)
Phacoemulsification	17.4% (n = 12)
Pterygium excision	2.9% (n = 2)
**Ablation depth (µm)**	18.0 (±6.9, n = 55)

**Table 2:** Mean values of corneal astigmatism, HOA and corneal thickness (at the center and at the thinnest point) for eyes with SND undergoing PTK. Significant reduction was observed in all follow-ups except in the corneal thickness at the thinnest point in the follow-up >1 year.

	Pre-Operatively	<1 Year	>1 Year	Total
Corneal Astigmatism (dpt)	4.49 ± 3.56	1.32 ± 1.19	1.92 ± 1.24	1.49 ± 1.20
n	37	33	12	37
*p*-value		<0.001	0.034	<0.001
HOA (µm)	1.32 ± 1.17	0.52 ± 0.49	0.53 ± 0.40	0.55 ± 0.50
n	35	31	10	34
*p*-value		<0.001	0.005	<0.001
Corneal thickness at the center (µm)	568.95 ± 46.01	533.55 ± 31.53	533.17 ± 31.94	535.11 ± 30.47
n	37	33	12	37
*p*-value		<0.001	0.007	<0.001
Corneal thickness at the thinnest point (µm)	550.00 ± 47.86	527.15 ± 31.61	526.83 ± 33.80	528.03 ± 31.66
n	37	33	12	37
*p*-value		<0.001	0.058	<0.001

**Table 3:** Pre- and post-operative correlation of BVCA (logMAR) with HOA, corneal density and K_max_ in eyes with SND.

		HOA	Densitometry		K_max_
			0–2 mm	2–6 mm	
before PTK	Pearson-Correlation with BCVA (logMAR)	0.372	0.595	0.536	0.380
	significance (1-sided)	0.014	<0.001	0.001	0.010
	n	35	34	34	37
after PTK	Pearson-Correlation BCVA (logMAR)	0.482	0.481	0.381	0.379
	significance (1-sided)	0.002	0.002	0.013	0.010
	n	35	34	34	37

**Table 4:** Complication rate for eyes with SND after PTK.

	<1 Year (n = 63 Follow-Ups)	>1 Year (n = 21 Follow-Ups)	Total (n = 69 Follow-Ups)
Haze	31.7% (n = 20)	19.0% (n = 4)	26.1% (n = 18)
Mild	22.2% (n = 14)	4.8% (n = 1)	15.9% (n = 11)
Moderate	9.5% (n = 6)	14.3% (n = 3)	10.1% (n = 7)
Strong	0%	0%	0%
KSP and/or Dry Eye	12.7% (n = 8)	33.3% (n = 7)	18.8% (n = 13)
Mild	9.5% (n = 6)	14.3% (n = 3) (n = 3)(n = 3)(n = 3)(n = 3)(n = 3)	10.1% (n = 7)
Moderate	3.2% (n = 2)	14.3% (n = 3)	7.2% (n = 5)
Strong	0%	4.8% (n = 1)	1.4% (n = 1)
Residuum	19.0% (n = 12)	42.9% (n = 9)	27.5% (n = 19)
Mild	11.1% (n = 7)	28.6% (n = 6)	17.4% (n = 12)
Moderate	6.3% (n = 4)	9.5% (n = 2)	7.2% (n = 5)
Strong	1.6% (n = 1)	4.8% (n = 1)	2.9% (n = 2)
Wound healing disorder	0%	0%	0%
Recurrence	1.6% (n = 1)	19.0% (n = 4)	7.2% (n = 5)
Re-PTK *			5.8% (n = 4)

* Due to Residuum or Recurrence.

## Data Availability

The data presented in this study are available on reasonable request from the corresponding author. The data are not publicly available due to privacy restrictions.
